# Safety and immunogenicity of two novel type 2 oral poliovirus vaccine candidates compared with a monovalent type 2 oral poliovirus vaccine in children and infants: two clinical trials

**DOI:** 10.1016/S0140-6736(20)32540-X

**Published:** 2021-01-02

**Authors:** Xavier Sáez-Llorens, Ananda S Bandyopadhyay, Christopher Gast, Tirza De Leon, Rodrigo DeAntonio, Jose Jimeno, Maria Isabel Caballero, Gabriela Aguirre, M Steven Oberste, William C Weldon, Jennifer L Konopka-Anstadt, John Modlin, Novilia S Bachtiar, Alan Fix, John Konz, Ralf Clemens, Sue Ann Costa Clemens, Ricardo Rüttimann

**Affiliations:** a Infectious Disease Department, Hospital del Niño Dr José Renán Esquivel, Panama City, Panama; b Sistema Nacional de Investigación, Senacyt, Panama; c Bill & Melinda Gates Foundation, Seattle, WA, USA; d PATH, Washington DC, USA; e Dartmouth Geisel School of Medicine, Hanover, NH, USA; f Cevaxin, Panama City, Panama; g Cevaxin, David, Chiriqui, Panama; h VaxTrials, Panama City, Panama; i Fighting Infectious Diseases in Emerging Countries, Miami, FL, USA; j Division of Viral Diseases, National Center for Immunization and Respiratory Diseases, Centers for Disease Control and Prevention, Atlanta, GA, USA; k PT Bio Farma, Bandung, Indonesia; l Global Research in Infectious Diseases, Rio de Janeiro, Brazil; m Institute for Global Health, Siena University, Siena, Italy

## Abstract

**Background:**

Continued emergence and spread of circulating vaccine-derived type 2 polioviruses and vaccine-associated paralytic poliomyelitis from Sabin oral poliovirus vaccines (OPVs) has stimulated development of two novel type 2 OPV candidates (OPV2-c1 and OPV2-c2) designed to have similar immunogenicity, improved genetic stability, and less potential to reacquire neurovirulence. We aimed to assess safety and immunogenicity of the two novel OPV candidates compared with a monovalent Sabin OPV in children and infants.

**Methods:**

We did two single-centre, multi-site, partly-masked, randomised trials in healthy cohorts of children (aged 1–4 years) and infants (aged 18–22 weeks) in Panama: a control phase 4 study with monovalent Sabin OPV2 before global cessation of monovalent OPV2 use, and a phase 2 study with low and high doses of two novel OPV2 candidates. All participants received one OPV2 vaccination and subsets received two doses 28 days apart. Parents reported solicited and unsolicited adverse events. Type 2 poliovirus neutralising antibodies were measured at days 0, 7, 28, and 56, and stool viral shedding was assessed up to 28 days post-vaccination. Primary objectives were to assess safety in all participants and non-inferiority of novel OPV2 day 28 seroprotection versus monovalent OPV2 in infants (non-inferiority margin 10%). These studies were registered with ClinicalTrials.gov, NCT02521974 and NCT03554798.

**Findings:**

The control study took place between Oct 23, 2015, and April 29, 2016, and the subsequent phase 2 study between Sept 19, 2018, and Sept 30, 2019. 150 children (50 in the control study and 100 of 129 assessed for eligibility in the novel OPV2 study) and 684 infants (110 of 114 assessed for eligibility in the control study and 574 of 684 assessed for eligibility in the novel OPV2 study) were enrolled and received at least one study vaccination. Vaccinations were safe and well tolerated with no causally associated serious adverse events or important medical events in any group. Solicited and unsolicited adverse events were overwhelmingly mild or moderate irrespective of vaccine or dose. Nearly all children were seroprotected at baseline, indicating high baseline immunity. In children, the seroprotection rate 28 days after one dose was 100% for monovalent OPV2 and both novel OPV2 candidates. In infants at day 28, 91 (94% [95% CI 87–98]) of 97 were seroprotected after receiving monovalent OPV2, 134 (94% [88–97]) of 143 after high-dose novel OPV2-c1, 122 (93% [87–97]) of 131 after low-dose novel OPV2-c1, 138 (95% [90–98]) of 146 after high-dose novel OPV2-c2, and 115 (91% [84–95]) of 127 after low-dose novel OPV2-c2. Non-inferiority was shown for low-dose and high-dose novel OPV2-c1 and high-dose novel OPV2-c2 despite monovalent OPV2 recipients having higher baseline immunity.

**Interpretation:**

Both novel OPV2 candidates were safe, well tolerated, and immunogenic in children and infants. Novel OPV2 could be an important addition to our resources against poliovirus given the current epidemiological situation.

**Funding:**

Fighting Infectious Diseases in Emerging Countries and Bill & Melinda Gates Foundation.

## Introduction

Global eradication of polio appeared to be close after declarations indicating eradication of wild-types 2 and 3,^1^ and circulation of wild-type 1 limited to Afghanistan and Pakistan.^2^ However, eradication remains a challenge with increasing incidence of paralytic poliomyelitis cases due to circulating vaccine-derived polioviruses, increasing from 71 cases in 2018, to 366 in 2019, and 739 in 2020 (as of Dec 3).^2^, ^3^ The situation has been exacerbated by the temporary suspension of supplementary immunisation activities due to the evolving COVID-19 situation.^4^ In settings with persistently low immunisation coverage, person-to-person transmission of circulating vaccine-derived polioviruses—which have reacquired neurovirulence through loss of the genetic attenuation—are responsible for the increasing emergence of new outbreaks.^5^, ^6^ WHO has designated type 2 circulating vaccine-derived polioviruses as a Public Health Emergency of International Concern.^7^

**Research in context**

**Evidence before this study**

Faced with an increasing number of individuals contracting paralytic poliomyelitis due to type 2 circulating vaccine-derived polioviruses, WHO declared a Public Health Emergency of International Concern in April, 2014. More genetically stable oral polioviruses for vaccines are urgently needed. As part of the consortium developing the type 2 oral poliovirus vaccines (OPV2), we did not do a literature search as there is only one previously published report on novel OPV2. As part of the clinical development of two novel live type 2 oral poliovirus vaccine (OPV2) candidates, we have previously reported a small phase 1 study and a larger phase 2 study in adults to confirm the safety, tolerability, and immunogenicity of both candidates, in which we also assessed viral shedding and showed improved genetic stability and the induction of intestinal immunity by the candidates.

**Added value of this study**

The target population for poliovirus outbreak response campaigns and thus that of novel OPV2 will be young children and infants. This is the first study, to our knowledge, that reports safety, tolerability, and immunogenicity in this target population of children aged 1–4 years and infants aged 18–22 weeks for polio outbreak response. We also showed that in infants, one or two doses of either novel OPV2 candidate (at doses representing the extremes of the range expected from production lots in storage), were safe, well tolerated, and immunogenic, and with similar immunogenicity to monovalent OPV2. In preliminary analyses, the novel OPV2 candidates displayed lower stool shedding rates 28 days after vaccination than those observed with monovalent OPV2 in a historical control study.

**Implications of all the available evidence**

In response to the WHO declaration that type 2 circulating vaccine-derived poliovirus outbreaks are a Public Health Emergency of International Concern and as part of the Global Polio Eradication Initiative response strategy, two novel OPV2 vaccines have been designed and engineered to be more genetically stable than Sabin monovalent OPV2. These novel OPV2 vaccines have been developed on an accelerated track for outbreak control. Data from this study have been used to support the selection of one candidate and the submission to the WHO Emergency Use Listings procedure for approval to allow distribution and use of the vaccine in new outbreaks as the first vaccine to ever be used under the Emergency Use Listings process as of Nov 13, 2020. The present data are the only information available to our knowledge to inform policy makers, regulators, and health-care providers on the use of the new vaccine in the age group considered most susceptible to poliovirus transmission.

The type 2 component from the oral poliovirus vaccine (OPV) has been withdrawn from routine use globally since May, 2016,^8^ leaving a growing cohort of children and infants reliant exclusively on doses of injected inactivated polio vaccines (IPVs) for immunity against type 2 poliovirus.^9^ Unlike OPV, IPV has a limited role in inducing primary intestinal mucosal immunity.^9^ Therefore, interruption of faecal–oral viral transmission in circulating vaccine-derived poliovirus outbreaks requires immunisation with live OPVs. However, emergence of type 2 circulating vaccine-derived polioviruses was associated with previous outbreak control activities with use of Sabin monovalent type 2 OPVs (OPV2).^3^ In response to the dilemma of using monovalent OPV2 for outbreak response activities, which risks further cases of new type 2 circulating vaccine-derived polioviruses, the Global Polio Eradication Initiative has supported the accelerated development of more genetically stable type 2 polioviruses for vaccines.^10^

A consortium established to ensure the development and research of such poliovirus vaccines has produced two candidates, novel OPV2-c1 and novel OPV2-c2, with further genetic modifications introduced to enhance the stability of the Sabin attenuations and substantially mitigate the risk of reversion to neurovirulence while maintaining immunogenicity.^11^, ^12^ Our previously reported phase 1 study showed the safety, immunogenicity, shedding, and stability of both novel OPV2 candidates in healthy adults, in conditions of biological containment.^13^

Following the preliminary assessment, and confirmation in larger adult studies of the safety of monovalent OPV2 and both novel OPV2 candidates,^14^ we did the present studies to confirm their safety and immunogenicity in fully immunised young children and in infants. As monovalent OPV2 was withdrawn from routine use globally in April and May, 2016, before novel OPV2 candidates were available, two studies were prospectively designed to be aligned as far as possible in terms of study centres, population, design, and analyses; the first to provide historical control data with monovalent OPV2 against which to compare novel OPV2 candidates assessed in the second study. We report the results of both studies, the first safety, tolerability, and immunogenicity data for the two novel OPV2 vaccine candidates in children and infants.

## Methods

### Study design and participants

We did two single-centre, multi-site, partly-masked, randomised studies in the Cevaxin Vaccination Center network (Panama City, Panama). We did a prospective historical control phase 4 study to provide baseline data with Sabin monovalent OPV2 in two sites in Panama City immediately before completion of global withdrawal of type 2-containing live poliovirus vaccines in May, 2016. The subsequent phase 2 study of the two novel OPV2 candidates was done in three Cevaxin sites, two in Panama City and one in David, Panama. The coprimary objectives were to assess safety in children and infants and immunogenicity in infants of both novel OPV2 vaccine candidates compared with data obtained from Sabin monovalent OPV2 vaccine in the historical control study.

Eligible participants in both studies were cohorts of healthy children and infants of either sex. Children were aged 1–4 years inclusive with a documented history of complete polio immunisation with either trivalent OPV or IPV. Infants were enrolled when aged 6 weeks to ensure they received three doses of bivalent OPV at 6, 10, and 14 weeks and one dose of IPV at 14 weeks to construct uniformly primed cohorts. Other inclusion and exclusion criteria are provided in the appendix (pp 2–4). In the phase 4 monovalent OPV2 study, children and infants were enrolled and vaccinated simultaneously. In the phase 2 novel OPV2 study, the child cohort was vaccinated first and the safety data assessed by an independent data and safety monitoring board before infants received low-dose and then high-dose novel OPV2 candidates.

Written informed consent was provided by the parents or guardians of all participants. Both study protocols were approved by the ethical review committee of the Hospital del Niño Dr José Renán Esquivel (Panama City, Panama).

### Randomisation and masking

In the historical control study, children received two doses of monovalent OPV2 28 days apart. All infants received one dose of monovalent OPV2 at 18–22 weeks of age, and a randomly selected subset (n=50) received a second dose 28 days later. In the novel OPV2 study, children received two high doses (10^6^ 50% cell culture infectious dose [CCID]_50_) of novel OPV2-c1 or novel OPV2-c2 28 days apart. At 18–22 weeks of age, infants received one low dose (10^5^ CCID_50_) or high dose (10^6^ CCID_50_) of novel OPV2-c1 or novel OPV2-c2, and randomly selected subsets (n=50) of each group received a second dose 28 days later.

Both studies used block randomisation with computer-generated lists (Assign Data Management and Biostatistics, Innsbruck, Austria). In the historical study, random assignment was 1:4 for one versus two doses until all two-dose participants were enrolled to allow faster completion. In the novel OPV2 study, random assignment was 1:1 for candidates within dose level, and 1:4 for one versus two doses until the 50 two-dose participants were enrolled. The data and safety monitoring board approved initiation of infant vaccination after reviewing 14 days of unblinded safety data from 50 children, and approved escalation from low dose to high dose in infants after reviewing 14 days of unblinded safety data from at least 50 infants given the low dose.

### Procedures

The vaccine used in the historical control study was Polio Sabin Mono Two (monovalent OPV2), a Sabin strain type 2 (P712, Ch, 2ab strain; GlaxoSmithKline Biologicals, Rixensart, Belgium; lot number DOP2A004AZ). One dose, consisting of two drops (0·1 mL) administered with a supplied dropper, nominally contained 10^5^^··7^ CCID_50_ of Sabin type 2 virus at release.

Both novel OPV2 vaccine candidates—novel OPV2-c1 and novel OPV2-c2—were manufactured by PT Bio Farma (Bandung, Indonesia), a global provider of WHO-prequalified Sabin OPVs, using established two-tiered Vero cell bank (master and working cell banks) and novel OPV2 seed systems (master and working virus seeds for each candidate). Each novel OPV2 candidate is an attenuated serotype 2 poliovirus derived from a modified Sabin 2 infectious clone propagated in Vero cells. Both candidate strains included different combinations of five distinct modifications of the Sabin-2 genome, including changes to the RNA sequence in the 5′ untranslated region of polio genome, the capsid protein coding region (P1), the non-structural protein 2C, and the polymerase 3D.^11^, ^12^ Only changes to polymerase 3D result in a change in the amino acid sequence. Each novel OPV2 candidate was administered as either a low dose in two drops (0·1 mL) containing 10^5^ CCID_50_ in infants using a supplied dropper, or a high dose containing 10^6^ CCID_50_ administered as 20 drops (1·0 mL) measured from a syringe to infants and children.

Regarding safety, after initial post-vaccination safety monitoring, parents completed electronic diary cards and were interviewed at each study visit. Diaries solicited systemic adverse events (appendix p 5) for 7 days after each vaccination. Unsolicited adverse events, serious adverse events, and important medical events were reported up to 28 days after each vaccination. Study investigators (XS-L, TDL, and RDA) considered causality for all adverse events.

Clinical laboratory assessments were done for children at days 0, 7, 28, and 56, and for infants at days 0, 7, and 28 in the one-dose groups, and days 0, 28, 35, and 56 in the two-dose groups. The same haematology laboratory assessments were done for both studies. Following the phase 1 adult study of the novel OPV2 candidates,^13^ additional laboratory assessments, notably aspartate aminotransferase, creatine phosphokinase, direct bilirubin, total bilirubin, and γ-glutamyl transferase, were done in the novel OPV2 study in addition to the routine assessments done in the monovalent OPV2 control study.

Serum samples obtained at days 0, 7, 28, and 56 were stored at –20°C or less for shipping to the Centers for Disease Control and Prevention laboratories (Atlanta, GA, USA) to measure polio type 2 neutralising antibodies using the WHO standard microneutralisation assay (WHO EPI GEN 93.9), adapted as previously described.^15^ Immunogenicity analyses were done contemporaneously in a blinded manner for both studies. Antibody titres are expressed as group median log_2_ titres with 95% CIs, proportions with a reciprocal titre of eight or greater (seroprotection rate), and proportions either becoming seroprotected when seronegative at baseline or displaying four-fold or greater increases in titres from baseline to post-vaccination (seroconversion rate). As some participants had high baseline titres, seroconversion was only calculated for those whose baseline titres allowed observation of a four-fold increase without being above the upper limit of quantitation (median log_2_ of 10·5).

Stool samples were collected once per day for 10 days and then once per week from days 14 up until 28 days after each vaccination for assessment of viral shedding and determination of genetic stability and potential neurovirulence of shed virus as previously described.^13^ Complete shedding and genetic stability data will be reported separately.

### Outcomes

Endpoints used to determine the primary safety objective in children and infants were the incidences of causally associated serious adverse events, severe adverse events (grade 3 according to Common Terminology Criteria for Adverse Events 4.03^16^), important medical events, and clinically relevant laboratory deviations. The primary immunogenicity endpoint was the poliovirus type 2 seroprotection rate 28 days after one standard dose of monovalent OPV2 or novel OPV2 (low or high dose) in the infant cohorts. Additional safety endpoints comprised incidence, severity, and causality of serious adverse events, solicited adverse events, unsolicited adverse events, important medical events, and laboratory deviations after one or two doses of monovalent OPV2 or novel OPV2 candidates. Secondary immunogenicity endpoints included median and geometric mean neutralising antibody titres, seroprotection rate, and seroconversion rates against poliovirus type 2 after one or two monovalent OPV2 or novel OPV2 doses in all children and infant participants, exclusive of the primary immunogenicity endpoint.

### Statistical analysis

The sample size of 50 participants in each of the three 1–4-year cohorts was intended to provide adequate safety data before infant immunisation; full follow-up of 45 participants (assuming ≤10% dropout) yielded a 90% probability of observing one or more adverse events of a given type, when the true rate is 5% or greater. For the primary immunogenicity endpoint, we assumed the seroprotection rate of one dose of monovalent OPV2 or novel OPV2 in infant cohorts would be 95% or more and selected the sample size for the historical control to enable a non-inferiority evaluation of the novel OPV2 candidates relative to monovalent OPV2 with a 10% margin for the risk difference assuming a 10% or less dropout rate. The target sample size was increased from 114 infants in the historical control study to 162 per candidate and dose amount in the novel OPV2 study due to the higher than anticipated dropout from the per-protocol population in the control study. Non-inferiority evaluation was based on the lower confidence bound of the Miettinen–Nurminen score CI, using a one-sided α of 0·025 for each novel OPV2 candidate and dose amount. For safety and secondary immunogenicity evaluations of two doses, 50 randomly selected participants were assigned to receive a second dose of each candidate.

The per-protocol population used for immunogenicity evaluations consisted of all eligible study participants who received their assigned immunisations as scheduled with no exclusion criterion, excluding those who received any therapy that could substantially affect their immune status, and was adapted by timepoint to allow participants to contribute data to per-protocol analyses until such a time as they became disqualified. All deviations and violations occurring in the study were reviewed before unblinding and locking of the novel OPV2 study database, using common criteria across studies to classify them as either minor or major. Safety evaluation was done in the total vaccinated population, which included all vaccinated participants according to vaccine received.

Except for primary endpoint comparisons, data were generally summarised descriptively, with count and percentage computed for categorical variables, paired with two-sided 95% exact CIs for immune responses and shedding rates, and the median and corresponding bootstrap-based two-sided 95% CI for log_2_ antibody titres, for log_10_ CCID_50_ per g of shed virus, and for day of peak viral shedding. For summaries of safety events, analyses were done on the participant level, with individuals included once under the maximum severity of a given event type. Missing data were not imputed. Miettinen–Nurminen CIs were used for rate differences. Multiplicity for the primary endpoint was addressed using a Bonferroni-style reduction from the selected one-sided family-wise type I error rate of 0·05 to 0·025 to account for multiple independent comparisons to the common control, and within candidate across dose amounts by predefining the testing sequence, permitting non-inferiority of the lower dose to be declared only if first achieved at the higher dose amount. Shedding was compared with the two-sided Fisher's exact test. SAS, version 9.3 was used for analyses.

These studies were registered with ClinicalTrials.gov, NCT02521974 and NCT03554798.

### Role of the funding source

Two authors (ASB, JM) were employees of the study funder and were involved in study design, data interpretation, and writing of the report, but had no role in data collection. All authors contributed to the study, had full access to all the data, and reviewed and approved the manuscript. The corresponding author had final responsibility for the decision to submit for publication.

## Results

**Figure 1:**
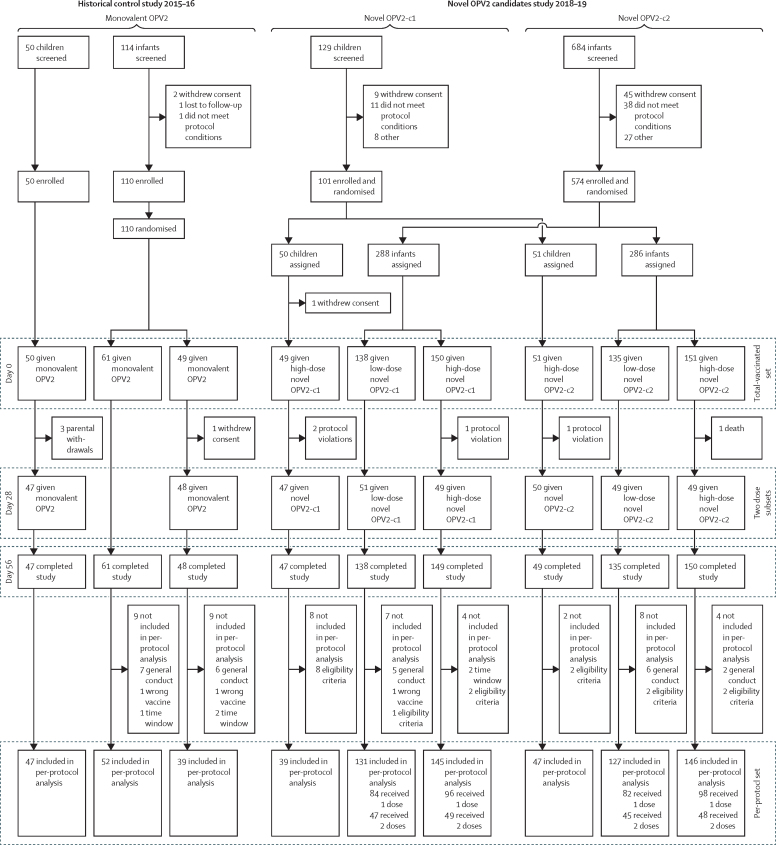
Trial profiles Per-protocol set used for immunogenicity analyses at day 28, and those additionally analysed at day 56 after two doses. OPV2=type 2 oral poliovirus vaccine.

The control phase 4 study took place between Oct 23, 2015, and April 29, 2016, and the subsequent phase 2 study between Sept 19, 2018, and Sept 30, 2019. We enrolled 151 children across both studies (table 1), with one child being withdrawn before vaccination (figure 1); children in the historical control study were generally older (mean 38·5 months [SD 14·2]) than the two groups in the novel OPV2 study (mean 29·8 months [12·0] in the OPV2-c1 group and 29·5 months [11·2] in the OPV2-c2 group). Polio vaccination histories reflected the withdrawal of trivalent OPV between studies; 45 (90%) of 50 children had received trivalent OPV in the control study, the remainder receiving IPV. In the novel OPV2 study, 10 (10%) of 101 children had received trivalent OPV, 69 (68%) had received bivalent OPV, and all had received at least one dose of IPV. The 684 infants enrolled across both studies had similar demographic characteristics across groups (table 1) and by design had the same polio vaccination history in both studies—three doses of bivalent OPV and one IPV.

**Table 1:** Baseline characteristics

		**Monovalent OPV2**		**Novel OPV2-c1**			**Novel OPV2-c2**		
		Children, standard dose (n=50)	Infant, standard dose (n=110)	Children, high dose (n=50)	Infant, low dose (n=138)	Infant, high dose (n=150)	Children, high dose (n=51)	Infant, low dose (n=135)	Infant, high dose (n=151)
Age, months or weeks*		38·5 (14·2)	19·0 (0·9)	29·8 (12·0)	18·7 (1·0)	18·5 (0·8)	29·5 (11·2)	18·6 (0·9)	18·4 (0·8)
Sex									
	Girls	29 (58%)	49 (45%)	25 (50%)	69 (50%)	79 (53%)	29 (57%)	61 (45%)	78 (52%)
	Boys	21 (42%)	61 (55%)	25 (50%)	69 (50%)	71 (47%)	22 (43%)	74 (55%)	73 (48%)
Weight, kg		15·2 (3·6)	7·2 (1·0)	13·2 (2·2)	7·2 (0·9)	7·1 (0·8)	12·9 (2·6)	7·4 (1·0)	7·2 (0·9)
Race									
	Mixed race	50 (100%)	108 (98%)	50 (100%)	135 (98%)	147 (98%)	51 (100%)	132 (98%)	151 (100%)
	Black	0	2 (2%)	0	0	1 (1%)	0	1 (1%)	0
	Central American Indian	0	0	0	3 (2%)	2 (1%)	0	1 (1%)	0
	Hispanic	0	0	0	0	0	0	0	0
	White	0	0	0	0	0	0	1 (1%)	0
Previous polio vaccinations†									
	Any bivalent OPV	0	..	36 (72%)	..	..	33 (65%)	..	..
	Any trivalent OPV	45 (90%)	..	6 (12%)	..	..	4 (8%)	..	..
	Any inactivated trivalent polio vaccine	5 (10%)	..	50 (100%)	..	..	51 (100%)	..	..

Data are mean (SD) or n (%). Doses were as follows: monovalent OPV2 was 10^5·7^ CCID_50_; low dose novel OPV2 was 10^5^ CCID_50_ and high dose novel OPV2 was 10^6^ CCID_50_. As noted in the methods, all infants received three doses of bivalent OPV and one dose of inactivated trivalent polio vaccine after enrolment, before monovalent OPV2 or novel OPV2 doses. OPV2=type 2 oral poliovirus vaccine. CCID=cell culture infectious dose. c1=candidate 1. c2=candidate 2.

* Mean age at first vaccination in months for children, weeks for infants.

† Vaccinations received by children before enrolment in the study: bivalent types 1 and 3 OPV; trivalent types 1, 2, and 3 OPV; and any inactivated trivalent polio vaccine.

In the historical control study, 50 children received one monovalent OPV2 vaccination, and 47 received a second dose 28 days later. Three children were withdrawn by their parents between the two study visits (figure 1). In the novel OPV2 study, 100 children received one novel OPV2 vaccination (47 received novel OPV2-c1 and 53 received novel OPV2-c2), and 97 received their second vaccinations (45 novel OPV2-c1 and 52 novel OPV2-c2), including two children who were randomly assigned to receive two doses of novel OPV2-c1 but who were given novel OPV2-c2 due to an administrative error. Both children were included in the appropriate novel OPV2-c2 group in the safety analyses, but not in the per-protocol immunogenicity analyses.

In the historical control study, 110 infants received a first dose of monovalent OPV2 and 48 received a second dose 28 days later (figure 1). In the novel OPV2 study, 574 infants received the first dose of their assigned vaccine, and 199 of 200 (between 49 and 51 per dose group of each candidate) received a second dose; one infant assigned to receive two doses of high-dose novel OPV2-c1 did not receive their second dose due to household contact with bivalent OPV after the first dose.

No child had a causally associated serious adverse event or important medical event after monovalent OPV2 or novel OPV2. Three children had serious adverse events leading to hospital admission: onset of pneumonia 25 days after a second monovalent OPV2 dose in the historical control study, mild bronchitis 13 days after a second high dose of novel OPV2-c1, and a soft tissue preauricular abscess 24 days after receiving high-dose novel OPV2-c2 (table 2). None were considered to be causally associated with vaccination.

**Table 2:** Occurrence of solicited adverse events within 7 days, and serious adverse events, important medical events, and unsolicited adverse events within 30 days of first or second vaccinations in children

		**Monovalent OPV2**		**Novel OPV2-c1 (high dose)**		**Novel OPV2-c2 (high dose)**	
		Dose 1 (n=50)	Dose 2 (n=47)	Dose 1 (n=47)	Dose 2 (n=45)	Dose 1 (n=53)	Dose 2 (n=52)
Serious adverse events		0	1 (2%)	0	1 (2%)	1 (2%)	0
	Vaccine-related serious adverse events	0	0	0	0	0	0
Important medical events		0	0	1 (2%)	0	0	0
	Vaccine-related important medical events	0	0	0	0	0	0
Solicited adverse events							
	Any	10 (20%)	9 (19%)	17 (36%)	12 (27%)	14 (26%)	12 (23%)
	Grade 1	9 (18%)	7 (15%)	13 (28%)	10 (22%)	8 (15%)	10 (19%)
	Grade 2	0	2 (4%)	3 (6%)	1 (2%)	3 (6%)	1 (2%)
	Grade 3	1 (2%)	0	1 (2%)	1 (2%)	3 (6%)	1 (2%)
	Causally associated (adverse reactions)	1 (2%)	0	5 (11%)	10 (22%)	4 (8%)	4 (8%)
Unsolicited adverse events							
	Any	37 (74%)	24 (51%)	35 (74%)	32 (71%)	41 (77%)	42 (81%)
	Causally associated	0	0	1 (2%)	0	0	0

No serious adverse events or important medical events were considered to be serious adverse reactions or important medical reactions (ie, causally associated with vaccination). OPV2=type 2 oral poliovirus vaccine.

Solicited adverse events were reported in 10 (20%) of 50 children after their first dose of monovalent OPV2 in the historical control study, and 17 (36%) of 47 children given first doses of novel OPV2-c1 and 14 (26%) of 53 children given first doses of novel OPV2-c2, in the second study. Rates were slightly lower after the second dose in each group (table 2). Most solicited adverse events, mainly consisting of transient loss of appetite, abnormal crying, irritability, and fever, were described as mild with few individuals having adverse events described as severe. Only one solicited adverse event was considered to be causally associated with monovalent OPV2, whereas 15 solicited adverse events after novel OPV2-c1 and eight solicited adverse events after novel OPV2-c2 were considered causally associated.

Most children were reported to have unsolicited adverse events within 4 weeks of any vaccination: 37 (74%) of 50 monovalent OPV2 recipients in the historical control study, 35 (74%) of 47 novel OPV2-c1 recipients, and 41 (77%) of 53 novel OPV2-c2 recipients after the first dose (table 2). Proportions were similar after novel OPV2 second doses, but lower after monovalent OPV2. One unsolicited adverse event considered to be causally associated with the vaccination was mild diarrhoea after one dose of novel OPV2-c1.

The death on day 25 of an infant admitted to hospital with severe pneumonia 7 days after one high-dose of novel OPV2-c2 was not considered to be causally associated with the vaccination. An infant admitted to hospital in the control study with moderate bronchiolitis after one monovalent OPV2 vaccination was considered a serious adverse event, and another infant with mild bronchiolitis after the second dose was an important medical event; neither was considered consistent with any causal association with vaccination. Nine serious adverse events after novel OPV2-c1 (low and high dose) and 13 serious adverse events after novel OPV2-c2 (low and high dose) (table 3) were mainly cases of community-acquired pneumonia or mild bronchiolitis, and none were causally associated with vaccination (appendix p 6).

**Table 3:** Occurrence of solicited adverse events within 7 days, and serious adverse events, important medical events, and unsolicited adverse events within 30 days of first or second vaccinations in infants

		**Monovalent OPV2**		**Novel OPV2-c1**				**Novel OPV2-c2**			
		Standard dose		Low dose		High dose		Low dose		High dose	
		Dose 1 (n=110)	Dose 2 (n=48)	Dose 1 (n=138)	Dose 2 (n=51)	Dose 1 (n=150)	Dose 2 (n=49)	Dose 1 (n=135)	Dose 2 (n=49)	Dose 1 (n=151)	Dose 2 (n=50)
Serious adverse events		1 (1%)	0	1 (1%)	1 (2%)	6 (4%)	1 (2%)	2 (1%)	1 (2%)	9 (6%)	1 (2%)
	Vaccine-related serious adverse events	0	0	0	0	0	0	0	0	0	0
Important medical events		0	1 (2%)	0	0	0	0	0	0	0	0
	Vaccine-related important medical events	0	0	0	0	0	0	0	0	0	0
Solicited adverse events											
	Any	29 (26%)	7 (15%)	37 (27%)	13 (25%)	49 (33%)	14 (29%)	42 (31%)	13 (27%)	52 (34%)	12 (24%)
	Grade 1	21 (19%)	6 (13%)	24 (17%)	6 (12%)	20 (13%)	6 (12%)	29 (21%)	10 (20%)	36 (24%)	10 (20%)
	Grade 2	6 (5%)	1 (2%)	12 (9%)	7 (14%)	28 (19%)	8 (16%)	12 (9%)	3 (6%)	15 (10%)	2 (4%)
	Grade 3	2 (2%)	0	1 (1%)	0	1 (1%)	0	1 (1%)	0	1 (1%)	0
	Causally associated (adverse reactions)	14 (13%)	5 (10%)	35 (25%)	12 (24%)	46 (31%)	11 (22%)	37 (27%)	11 (22%)	45 (30%)	12 (24%)
Unsolicited adverse events											
	Any	74 (67%)	33 (69%)	70 (51%)	30 (59%)	94 (63%)	33 (67%)	89 (66%)	29 (59%)	96 (64%)	30 (60%)
	Causally associated	1 (1%)	0	0	0	2 (1%)	0	0	0	0	1 (2%)

No serious adverse events or important medical events were considered to be serious adverse reactions or important medical reactions (ie, causally associated with vaccination). OPV2=type 2 oral poliovirus vaccine.

Solicited adverse events were reported at slightly higher rates in infants than in children with a trend for higher rates after novel OPV2 than after monovalent OPV2. After the first dose of monovalent OPV2, 29 (26%) of 110 infants had a solicited adverse event, and seven (15%) of 48 had a solicited adverse event after their second dose. 14 (13%) of 110 had solicited adverse events considered causally associated with the first dose of monovalent OPV2 and five (10%) of 48 had solicited adverse events considered causally associated with the second dose of monovalent OPV2 (table 3). Most frequent solicited adverse events were mild or moderate cases of abnormal crying, irritability, and vomiting; only two severe solicited adverse events were reported after the first dose. Rates of solicited adverse events were 25–33% after first or second doses of low-dose or high-dose novel OPV2-c1 and 24–34% after low-dose or high-dose novel OPV2-c2 (table 3). Most were transient, mild, or moderate cases of abnormal crying, vomiting, irritability, and fever, and 22–30% across groups of first or second doses of either novel OPV2 candidate were considered causally associated. No cases of severe fever (>39°C) were reported. Most infants (51–69% across groups) were reported with mild or moderate unsolicited adverse events (table 3). Four infants had mild unsolicited adverse events considered to be possibly causally associated with vaccination: anaemia after monovalent OPV2 (first dose), diarrhoea and nasopharyngitis after novel OPV2-c1 (both first dose), and fever after novel OPV2-c2 (second dose).

No consistent pattern of grade 3 or 4 clinical laboratory abnormalities was observed at any point, nor any clinically relevant abnormalities or changes in laboratory assessments in children or infants after vaccination in either study (appendix p 7). Notably, the elevated amounts of liver enzymes and creatine phosphokinase observed in some adult participants of the phase 1 study^14^ were not present in children or infants. For other clinical parameters, some grade 1 or 2 abnormalities were present at baseline, before vaccination, and some remained so during the post-vaccination period, but most remained within the normal ranges. More than 90% of children and infants had atypical lymphocyte counts at baseline or day 7, but these were mainly mild (grade 1) and did not persist throughout the study. The other most frequent observations were anaemia (low haemoglobin counts) and low white blood cell counts, which were also observed before vaccination, and were transient.

As expected, given their immunisation history, children had high baseline seroprotection rates against type 2 poliovirus: 100% in the historical control monovalent OPV2 group, and 100% in the novel OPV2-c1 group and 94% in the novel OPV2-c2 group (table 4). In the novel OPV2 study, median titres had increased to the upper limit of quantitation by day 7 in both novel OPV2 groups, achieving 100% seroprotection rate in both candidate groups at days 28 and 56. Due to their high baseline titres, it was only possible to assess seroconversion in nine children in the control study, six (67%) of whom seroconverted by day 28. This rate did not increase by day 56 after a second dose. Seroconversion rates were higher after novel OPV2 than after monovalent OPV2, although this result is notably confounded with the number, type, and time since last type 2 vaccination, and possible environmental exposure of monovalent OPV2 vaccinees to type 2 vaccine before type 2 OPV withdrawal. Evaluable seroconversion rates were 94–100% after one or two doses in novel OPV2 candidate groups.

**Table 4:** Median poliovirus neutralising antibody titres, seroprotection rates, and seroconversion rates in the per-protocol population of children

	**Monovalent OPV2**	**Novel OPV2-c1**	**Novel OPV2-c2**
**Median poliovirus neutralising antibody titres**			
Day 0, baseline	49, 10·50 (9·83–10·50)	41, 9·17 (7·50–9·83)	47, 8·50 (7·17–9·50)
Day 7, after dose 1	46, 10·50 (10·17–10·50)	39, 10·50 (10·50–10·50)	47, 10·50 (10·50–10·50)
Day 28, after dose 1	46, 10·50 (10·50–10·50)	37, 10·50 (10·50–10·50)	47, 10·50 (10·50–10·50)
Day 56, after dose 2	46, 10·50 (10·50–10·50)	37, 10·50 (10·50–10·50)	46, 10·50 (10·50–10·50)
**Seroprotection rates**			
Day 0, baseline	49/49 (100%); 93–100	41/41 (100%); 91–100	44/47 (94%); 83–99
Day 7, after dose 1	46/46 (100%); 92–100	36/37 (97%); 86–100	47/47 (100%); 93–100
Day 28, after dose 1	46/46 (100%); 92–100	37/37 (100%); 91–100	47/47 (100%); 93–100
Day 56, after dose 2	46/46 (100%); 92–100	37/37 (100%); 91–100	47/47 (100%); 93–100
Seroconversion rates*			
Day 7, after dose 1	3/8 (38%); 9–76	11/17 (65%); 38–86	19/24 (79%); 58–93
Day 28, after dose 1	6/9 (67%); 30–93	16/17 (94%); 71–100	23/24 (96%); 79–100
Day 56, after dose 2	6/9 (67%); 30–93	17/17 (100%); 81–100	22/23 (96%); 78–100

Data are n, median log_2_ (95% CI), or n/N (%); 95% CI. Doses were as follows: monovalent OPV2 was 10^5^^·^^7^ CCID_50_; novel OPV2 was 10^6^ CCID_50_. OPV2=type 2 oral poliovirus vaccine. CCID=cell culture infectious dose. For titres, in all cases, the use of 2·50 should be interpreted as 2·50 or less and the use of 10·50 should be interpreted as 10·50 or greater.

* Seroconversion was only measured in those participants whose initial antibody titre allowed observation of a four-fold increase.

The predefined non-inferiority criterion was met for both the low-dose and high-dose novel OPV2-c1 in infants, and for the high-dose novel OPV2-c2 (table 5). We did not observe non-inferiority of low-dose novel OPV2-c2 as the lower bound of the CI was –10·6% (ie, just below the –10% non-inferiority margin). A second novel OPV2 dose increased seroprotection to 98% in monovalent OPV2 and low-dose novel OPV2 groups, and 100% in high-dose novel OPV2 groups (table 5).

**Table 5:** Median poliovirus neutralising antibody titres, seroprotection rates, and seroconversion rates in the per-protocol population of infants

	**Monovalent OPV2**	**Novel OPV2-c1**		**Novel OPV2-c2**	
	Standard dose	Low dose	High dose	Low dose	High dose
**Median poliovirus neutralising antibody titres**					
Day 0, baseline	102, 3·83 (3·50–3·92)	131, 3·50 (3·17–3·83)	146, 3·17 (3·09–3·50)	129, 3·50 (3·50–3·83)	147, 3·50 (3·17–3·83)
Day 7*, after dose 1	56, 6·67 (5·83–7·50)	84, 5·83 (5·00–6·50)	97, 5·17 (4·17–6·17)	84, 5·83 (4·83–6·50)	97, 6·50 (5·17–6·83)
Day 28, after dose 1	97, 10·50 (10·50–10·50)	131, 10·50 (10·50–10·50)	143, 10·50 (10·34–10·50)	127, 10·17 (9·83–10·50)	146, 10·17 (10·17–10·50)
Day 56*, after dose 2	40, 10·50 (10·50–10·50)	46, 10·50 (10·17–10·50)	48, 10·34 (10·17–10·50)	44, 9·83 (9·34–10·34)	48, 10·17 (9·83–10·50)
**Seroprotection rates**					
Day 0, baseline	77/102 (76%); 66–84	88/131 (67%); 58–75	85/146 (58%); 50–66	88/129 (68%); 59–76	103/147 (70%); 62–77
Day 7*, after dose 1	52/56 (93%); 83–98	75/84 (89%); 81–95	85/97 (88%); 79–93	72/84 (86%); 76–92	84/97 (87%); 78–93
Day 28, after dose 1	91/97 (94%); 87–98	122/131 (93%); 87–97	134/143 (94%); 88–97	115/127 (91%); 84–95	138/146 (95%); 90–98
Non-inferiority comparison†	1 (ref)	−0·7% [−7·4 to 6·7]‡	−0·1% [−6·4 to 7·2]‡	−3·3% [−10·6 to 4·5]§	−0·7% [−5·3 to 7·9]‡
Day 56*, after dose 2	39/40 (98%); 87–100	45/46 (98%); 89–100	48/48 (100%); 93–100	43/44 (98%); 88–100	48/48 (100%); 93–100
**Seroconversion rates**¶					
Day 7*, after dose 1	40/56 (71%); 58–83	45/80 (56%); 45–67	54/95 (57%); 46–67	44/83 (53%); 42–64	57/97 (59%); 48–69
Day 28, after dose 1	88/96 (92%); 84–96	108/126 (86%); 78–91	121/141 (86%); 79–91	102/126 (81%); 73–87	126/146 (86%); 80–91
Day 56*, after dose 2	38/39 (97%); 87–100	44/45 (98%); 88–100	47/48 (98%); 89–100	38/44 (86%); 73–95	48/48 (100%); 93–100

Data are n, median log_2_ (95% CI), or n/N (%); 95% CI, or difference (95% CI). Doses were as follows: monovalent OPV2 was 10^5^^·^^7^ CCID_50_; low dose novel OPV2 was 10^5^ CCID_50_ and high dose novel OPV2 was 10^6^ CCID_50_. OPV2=type 2 oral poliovirus vaccine. CCID=cell culture infectious dose. For titres, in all cases the use of 2·50 should be interpreted as 2·50 or less and the use of 10·50 should be interpreted as 10·50 or greater.

* Day 7 rates are only shown for participants who received one vaccination, day 56 rates only for those who received two vaccinations.

† Difference between novel OPV2 compared with monovalent OPV2.

‡ Non-inferiority criterion met in which the 95% CI of difference between monovalent OPV2 and novel OPV2 was within 10%.

§ Non-inferiority could not be seen.

¶ Seroconversion was only measured in those participants whose initial antibody titre allowed observation of a four-fold increase.

Infants in the historical control study had a higher baseline seroprotection rate (76% [95% CI 66–84], n=102) than either novel OPV2 group (63% [57–68], n=277 for novel OPV2-c1 and 69% [63–75], n=276 for novel OPV2-c2; table 5). On day 28, after one dose of monovalent OPV2, the seroprotection rate increased to 94% (95% CI 87–98) with seroconversion in 88 (92%) of 96 evaluable participants. At day 28, rates were 91–95% after receiving one low or high dose of either novel OPV2 candidate.

At day 56, after two doses of monovalent OPV2, the seroconversion rate was 97% (95% CI 87–100). The high-dose novel OPV2-c1 group had a conversion rate of 98% (89–100) and the high-dose novel OPV2-c2 group had a rate of 100% (93–100); both groups received similar nominal viral doses as the monovalent OPV2 group. Seroconversion in the low-dose novel OPV2 groups, with ten-fold less virus, were 98% (88–100) for novel OPV2-c1, and 86% (73–95) for novel OPV2-c2.

**Figure 2:**
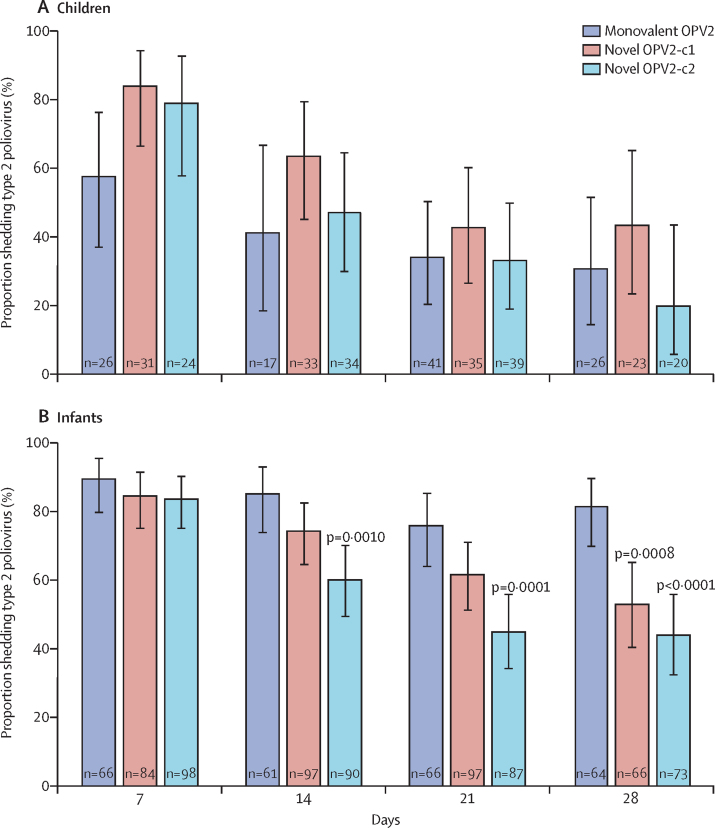
Type 2 shedding in children and infants after monovalent OPV2 and high-dose novel OPV2 administration Proportions (with 95% CI) of children (A) and infants (B) shedding poliovirus type 2 (PCR-positive stools) 7, 14, 21, and 28 days after vaccination with monovalent OPV2 or high-dose novel OPV2 candidates. Shedding was compared with the two-sided Fisher's exact test. p values indicated when novel OPV2 data were significantly different versus monovalent OPV2. OPV2=type 2 oral poliovirus vaccine.

Analysis of poliovirus in stools is ongoing, but data are available after the first high dose in children and infants. Children had significantly higher peak shedding titres after either novel OPV2 candidate than monovalent OPV2 (appendix p 8). In infants who received monovalent OPV2 or either high-dose novel OPV2 candidate, 99% shed type 2 virus with similar peak median shedding titres and times to peak shedding in these groups: median log_10_ CCID_50_ after monovalent OPV2 was 3·16 (95% CI 3·03–3·75) compared with 3·28 (3·03–3·69) after novel OPV2-c1 and 3·06 (2·91–3·44) after novel OPV2-c2 (appendix p 8). Proportions shedding at day 7 were similar for monovalent OPV2 and both novel OPV2 candidates, but a significantly lower rate of shedding following either novel OPV2 compared with monovalent OPV2 emerged by day 28 in infants (figure 2).

## Discussion

These two separate studies in previously vaccinated children and in infants provide support for the safety and acceptable tolerability of low and high doses of two novel OPV2 candidates, similar to the licensed monovalent OPV2 vaccine in infants, and of high doses in young children. All vaccines displayed acceptable reactogenicity profiles with mainly mild and transient systemic adverse events. Serious or severe adverse events were infrequent and not considered causally associated with vaccination. The prevalence of adverse events was generally similar in the monovalent OPV2 historical control study and the novel OPV2 study with the two novel candidate vaccines. The death of one infant who was admitted to hospital after developing severe pneumonia was not considered to be associated with the study vaccine or procedures. Higher association of causality with novel OPV2 vaccines than monovalent OPV2 might be due to the open-label nature of these studies comparing licensed and investigational vaccines.

The predefined non-inferiority criterion for seroprotection in infants at day 28 was met for most comparisons of both low-dose and high-dose novel OPV2 candidates related to the monovalent OPV2 vaccine after one dose. The exception was the low-dose novel OPV2-c2, which just fell outside of the 10% criterion (–10·6%). All vaccinations resulted in high seroprotection, with seroconversion evident in most participants whose baseline titres allowed evaluation.

A growing global cohort of children immunised only with bivalent OPV and IPV have minimal intestinal immunity against type 2 poliovirus.^17^ As use of monovalent OPV2 to interrupt transmission of type 2 circulating vaccine-derived polioviruses might itself induce further emergence of them in some settings, the Global Polio Eradication Initiative has supported the accelerated development of more genetically stable OPV2 vaccines. The two tested novel OPV2 candidates, which have been developed in parallel, are the first new OPV strains in advanced clinical development in approximately 60 years. Both candidates have been engineered to improve the genetic stability of the attenuation sites of the poliovirus and so minimise the risk of reversion to neurovirulence.^11^, ^12^

Safety and immunogenicity of both candidates shown in a phase 1 study in adults, done in strict containment conditions to avoid possible environmental contamination with the novel viruses,^13^ has been confirmed by a larger non-contained study in adults.^14^ Both studies also provided a preliminary assessment of the genetic stability in virus shed in stools, sufficient to pave the way for the present study in children and infants.

Novel OPV2 is not intended for routine vaccination. Currently, children in most developing country settings with bivalent OPV and IPV in the routine immunisation schedule will be expected to have high systemic and intestinal immunity against poliovirus types 1 and 3, with one dose of IPV providing only partial humoral and minimal intestinal protection against poliovirus type 2,^9^ making them susceptible to type 2 circulating vaccine-derived polioviruses. In this context, it is encouraging to observe robust protective type 2 immune responses similar to the historical controls following doses of each novel OPV2 candidate mimicking the range of doses expected from newly produced lots to end of expiration lots, although low-dose novel OPV2-c2 did not meet the strict non-inferiority criterion.

An unavoidable limitation of this study was the necessity to establish control data in children and infants who received monovalent OPV2 before its global withdrawal, except for emergency use, in May, 2016.^8^ Final selection of the vaccine candidate and manufacture of the novel OPV2 were completed in 2018, so a direct concurrent, comparison was not possible. The historical control study was proactively designed and done to provide control data with monovalent OPV2 before the withdrawal, using the same study centres, similar protocols, and simultaneous serological laboratory assessments, among others, to minimise confounding factors.

Comparison of immunogenicity and viral shedding data among cohorts of children aged 1–5 years is generally not possible because of the different immunisation backgrounds of the two studies, with more monovalent OPV2 recipients having received trivalent OPV than novel OPV2 cohorts. However, data from comparable infant cohorts indicated that the viral shedding rate, an indicator of vaccine-induced intestinal immunity, is similar in peak quantity proximal to vaccination, with a lower shedding rate than monovalent OPV2 emerging by 28 days post-vaccination.^18^, ^19^ These data will be assessed further when analyses of genetic stability and neurovirulence of shed virus are available. Data from participants who received two doses of the vaccine candidates might also inform on the induction of intestinal immunity.^9^ Data from the phase 1 and phase 2 adult studies suggested that the objective of improved genetic stability to decrease reversion to neurovirulence has been achieved, but data from larger studies will be necessary to confirm this observation.^13^, ^14^

These findings showing that both novel OPV2 candidate vaccines are safe, well tolerated, and immunogenic in young children and infants, together with manufacturing and epidemiological considerations, contributed to selection of novel OPV2-c1 as the first new vaccine to be listed under the WHO Emergency Use Listing procedure for use in type 2 circulating vaccine-derived poliovirus outbreaks.^20^ Further studies including a phase 2 trial with novel OPV2-c1 and bivalent OPV (NCT 04579510) and a phase 3 trial in The Gambia (WHO PACTR202010705577776) are planned. Our data are essential to inform the ongoing policy and regulatory assessments to enable the proposed roll-out of novel OPV2-c1 in 2020 and 2021.^21^, ^22^

## Data sharing

Data for this study will be made available to others in the scientific community upon request after the ongoing phase 3 study of the selected vaccine candidate has been completed and the scientific data from the development of both candidates have been fully published. Standard criteria for making data available for valid research projects will be used, following application by suitably qualified researchers. For data access, please contact the Gates Foundation at openaccess@gatesfoundation.org.
