# Supplementary material for: Safety and immunogenicity of two novel type 2 oral poliovirus vaccine candidates compared with a monovalent type 2 oral poliovirus vaccine in children and infants: two clinical trials
Source: Lancet. 2021 Jan 2;397(10268):27–38. doi: 10.1016/S0140-6736(20)32540-X (PMC7811205; doi:10.1016/S0140-6736(20)32540-X)
Supplement: Supplementary appendix [file mmc1.pdf]

THE LANCET

Supplementary appendix

This appendix formed part of the original submission and has been peer reviewed. We post it as supplied by the authors.

Supplement to: Sáez-Llorens X, Bandyopadhyay AS, Gast C, et al. Safety and immunogenicity of two novel type 2 oral poliovirus vaccine candidates compared with a monovalent type 2 oral poliovirus vaccine in children and infants: two clinical trials. *Lancet* 2020; published online Dec 9. http://dx.doi.org/10.1016/S0140-6736(20)32540-X.

Sáez-Llorens et al, Lancet 2020

Supplementary Appendix

Inclusion criteria for both studies page 2.

Exclusion criteria for both studies page 3.

Table 1: Solicited adverse events and grading for intensity page 5.

Table 2: Serious adverse events (SAE) in infants page 6.

Table 3: Grade 3 or 4 clinical laboratory abnormalities page 7.

Table 4: Shedding data in children and infants page 8.

Inclusion criteria for participants in the monovalent OPV2 and novel OPV2 studies:

All potential participants had to meet all of the following criteria to included in the monovalent OPV2 and novel OPV2 studies:

1. For children enrolled at 1 to 4 years of age, in the monovalent OPV2 study children who had previously received 3 or 4 doses of trivalent OPV or IPV, and for the novel OPV2 study children who had been fully vaccinated according to Panamanian Ministry of Health recommendations with OPV and/or IPV.

2. For both studies, for infants enrolled at 6 weeks of age with birth weight >2,500 to be eligible to continue into the experimental phase of the study infants were to have been vaccinated with 3 doses of bivalent OPV and one dose of IPV prior to administration of the study vaccine at 18–22 weeks of age. The last polio vaccine must have been administered at least 4 weeks prior to the first dose of study vaccine.

   An additional instruction in the novel OPV2 study concerning the need to take into account visit windows for enrolment (age 6 weeks, -1 or + 2 weeks) and subsequent OPV vaccination windows (± 1 week) was provided. This criterion also instructed the Investigator to replace any infants who had not completed the three routine vaccination visits and to encourage parents/guardians to complete the primary series.)

3. All participants were required to be healthy with no obvious medical conditions like immunodeficiency diseases, severe congenital malformations, severe neurological diseases or any other diseases that require high doses of corticosteroids or immunotherapies, that preclude the participant to be in the study as established by the medical history and physical examination.

4. Written informed consent was obtained from 1 or 2 parent(s) or legal guardian(s) as per national regulations.

Exclusion criteria for participants in the monovalent OPV2 and novel OPV2 studies:

Potential participants who met any of the following criteria were excluded from the monovalent OPV2 and novel OPV2 studies:

1. For all participants, the presence of anyone under 10 years of age in the participant's household (living in the same house or apartment unit) who did not have complete "age appropriate" vaccination status with respect to poliovirus vaccines at the time of study vaccine administration. For household members younger than 18 months "age appropriate" vaccination is at least three (3) doses of IPV. For the novel OPV2 study household members between 18 months and 10 years "age appropriate" vaccination is at least three doses of IPV or trivalent OPV plus one booster dose of any anti-polio vaccine.
2. For all participants in the novel OPV2 study, having a member of the participant's household (living in the same house or apartment unit) who was under 6 months of age at the moment of study vaccine administration.
3. For all participants, having a member of the participant's household (living in the same house or apartment unit) who had received OPV in the previous 3 months before study vaccine administration.
4. For children in either study: receipt of polio vaccines within the 3 months prior to the administration of the study vaccine (number of previous polio vaccine doses to be documented), or any other vaccine 4 weeks before study entry.
5. For children in the novel OPV2 study: any participating children attending day care or pre-school during their participation in the study until one month after their last novel OPV2 administration.
6. For infants in either study: any receipt of polio vaccines prior to administration of the study vaccine other than three doses of bivalent OPV and one dose of IPV.
7. Any confirmed or suspected immunosuppressive or known immunodeficient condition including human immunodeficiency virus (HIV) infection in the potential participant or any member of the participant's household.
8. Family history of congenital or hereditary immunodeficiency.

9. Major congenital defects or serious uncontrolled chronic illness (neurologic, pulmonary, gastrointestinal, hepatic, renal, or endocrine).
10. Known allergy to any component of the study vaccines or to any antibiotics that share molecular composition with the novel OPV2 vaccines. (The specifics of antibiotics that share molecular composition with the novel OPV2 vaccines was added for the novel OPV2 study.)
11. Uncontrolled coagulopathy or blood disorder contraindicating intramuscular vaccinations.
12. Administration of immunoglobulins and/or any blood products since birth or planned administration during the study period.
13. Acute severe febrile illness at day of vaccination deemed by the Investigator to be a contraindication for vaccination (the child could be included at a later time if within age window and all inclusion criteria were met).
14. Any participant who, in the opinion of the Investigator, was unlikely to comply with the protocol or was inappropriate to be included in the study for the safety or the benefit-risk ratio of the participant.

Table 1. Solicited adverse events and intensity grading:

Solicited Adverse Event	Intensity grade	Parameter
**Fever**	0	< 37·5°C
	1	37·5°C to 38·0°C
	2	38·1°C to 39·0°C
	3	> 39·0°
**Vomiting**	0	None
	1	1 episode per 24 hours
	2	2–5 episodes per 24 hours
	3	≥ 6 episodes per 24 hours or requiring parenteral hydration
**Abnormal crying**	0	None
	1	< 1 hour
	2	1–3 hours
	3	> 3 hours
**Drowsiness**	0	None
	1	Sleepier than usual or less interested in surroundings
	2	Not interested in surroundings or did not wake up for a feed/meal
	3	Sleeping most of the time or difficult to wake up
**Loss of appetite**	0	None
	1	Eating less than normal
	2	Missed 1 or 2 feeds/meals completely
	3	Refuses ≥ 3 feeds/meals or refuses most feeds/meals
**Irritability**	0	None
	1	Easily consolable
	2	Requiring increased attention
	3	Inconsolable

Table 2. Serious adverse events (SAE) in infants within 30 days of vaccination in the two studies.

Vaccine	Vaccination no.	Low dose	High dose
**Monovalent OPV2**	**1**	-	1 bronchiolitis
	**2**	-	1 bronchiolitis
**Novel OPV2-c1**	**1**	1 pneumonia	3 pneumonia<br>3 bronchiolitis
	**2**	1 pneumonia	1 bronchiolitis
**Novel OPV2-c2**	**1**	1 bronchiolitis<br>1 congenital cortical blindness	2 pneumonia<br>5 bronchiolitis<br>1 virosis<br>1 urinary tract infection
	**2**	1 Pneumonia	1 bronchiolitis

No SAE was considered to be causally associated with vaccination and all resolved

Table 3. Clinically relevant (Grade 3 or 4) laboratory abnormalities up to 28 days after vaccination in children and infant TV populations of the two studies.

Laboratory assessment		Historical control study		Novel OPV2 study					
	Vaccine	**Monovalent OPV2**		**Novel OPV2-c1**			**Novel OPV2-c2**		
	Group	Children	Infants	Children	Infants		Children	Infants	
	Dosage	-	-	High	Low	High	High	Low	High
	N =	48	102	45	138	150	52	134	151
**Participants with any clinically relevant change after Day 0**		1 (2)	6 (6)	24 (53)	9 (7)	10 (7)	31 (62)	4 (3)	19 (13)
**APTT**	n (%)	0 (0)	0 (0)	0 (0)	0 (0)	0 (0)	0 (0)	0 (0)	1 (1)
**ALT**	n (%)	0 (0)	0 (0)	0 (0)	0 (0)	1 (1)	0 (0)	0 (0)	0 (0)
**AST**	n (%)	nd	0 (0)	0 (0)	0 (0)	1 (1)	0 (0)	0 (0)	0 (0)
**CPK**	n (%)	nd	nd	0 (0)	0 (0)	0 (0)	1 (2)	0 (0)	0 (0)
**Creatinine**	n (%)	0 (0)	0 (0)	0 (0)	0 (0)	0 (0)	0 (0)	0 (0)	0 (0)
**Fibrinogen**	n (%)	0 (0)	0 (0)	0 (0)	0 (0)	0 (0)	0 (0)	0 (0)	0 (0)
**GGT**	n (%)	nd	nd	0 (0)	0 (0)	0 (0)	0 (0)	0 (0)	0 (0)
**Haemoglobin**	n (%)	1 (2)	5 (5)	0 (0)	5 (4)	6 (4)	2 (4)	3 (2)	12 (8)
**Lymphocytes**	n (%)	0 (0)	0 (0)	8 (18)	3 (2)	2 (1)	12 (23)	0 (0)	1 (1)
**Neutrophils**	n (%)	1 (2)	0 (0)	0 (0)	5 (4)	1 (1)	5 (10)	0 (0)	1 (1)
**Platelets**	n (%)	0 (0)	2 (2)	2 (4)	0 (0)	2 (1)	2 (4)	1 (1)	5 (3)
**Total bilirubin**	n (%)	nd	nd	0 (0)	0 (0)	0 (0)	0 (0)	0 (0)	0 (0)
**White blood cell count**	n (%)	0 (0)	0 (0)	5 (11)	2 (1)	2 (1)	9 (17)	0 (0)	5 (3)

Only laboratory parameters that can be graded using the CTCAE scale are shown.

nd = not determined. APTT = activated partial thromboplastin time; ALT = alanine aminotransferase; AST = aspartate aminotransferase; CPK = creatine phosphokinase; GGT = gamma-glutamyl transferase.

Table 4. Poliovirus shedding after the first dose in those not shedding type 2 at baseline.

		Historical control study	Novel OPV2 study – High dose [a]	
**Children**				
		**Monovalent OPV2**	**Novel OPV2-c1**	**Novel OPV2-c2**
	N =	48	46	51
Participants shedding (Type 2 PCR+)	**n** (%) [95% CI]	**44** (92) [80–98]	**43** (93) [82–99]	**50** (98) [90–100]
Median Peak Log_{10} $CCID_{50}$/g (95% CI)		**2·77** (2·75–2·81)	**5·00** [b] (4·25–5·25)	**4·75** [b] (4·41–5·03)
Median time to peak shedding (days) (95% CI)		**6.5** (5·0–9·0)	**5.0** (4·0–5·0)	**3.0** (2·5–5·0)
**Infants – High dose novel OPV2**				
		**Monovalent OPV2**	**Novel OPV2-c1**	**Novel OPV2-c2**
	N =	91	140	141
Participants shedding (Type 2 PCR+)	n (%) [95% CI]	**90** (99) [94–100]	**139** (99) [96–100]	**140** (99) [96–100]
Median Peak Log_{10} $CCID_{50}$/g (95% CI)		**3·16** (3·03–3·75)	**3·28** (3·03–3·69)	**3·06** (2·91–3·44)
Median time to peak shedding (days) (95% CI)		**6·0** (5·0–7·0)	**6·0** (5·0–7·0)	**5·0** (4·0–6·0)

a: Monovalent OPV2 = $10^{5·7}$ $CCID_{50}$; high dosage novel OPV2 = 10^6 $CCID_{50}$

b: p < 0·0001 vs. monovalent OPV2
